# Enhanced Uptake of FDG in PET/CT After the Use of Bone Wax During Sternotomy

**DOI:** 10.1055/a-2796-6952

**Published:** 2026-02-19

**Authors:** Michael Jüptner, Bernd Panholzer, Anette Friedrichs, Gregor Warnecke, Jochen Cremer, Alexander Thiem, Ulf Lützen, Jan Schöttler

**Affiliations:** 1 Department of Nuclear Medicine, Molecular Diagnostic Imaging and Therapy, University Hospital Schleswig-Holstein, Campus Kiel, Kiel, Germany; 2 Department of Cardiac Surgery, University Hospital Schleswig-Holstein, Campus Kiel, Kiel, Germany; 3 Department of Internal Medicine I, Infectious Diseases, University Hospital Schleswig-Holstein, Campus Kiel, Kiel, Germany

**Keywords:** FDG, PET/CT, bone wax, hemostasis, sternotomy, inflammation

## Abstract

**Objective:**

Physiologic healing processes and foreign body reactions can mimic infective conditions in ^18^ F-FDG-PET/CT for the detection of deep sternal wound infections. To date, nothing is known about the metabolic presentation of surgically applied bone wax to the sternum for hemostasis during sternotomy in ^18^ F-FDG-PET/CT imaging. Therefore, this study aims to assess the sternal FDG uptake after the application of bone wax during sternotomy.

**Methods:**

A total of 25 patients with a history of cardiac surgery (1.3–5.5 years ago) were examined by ^18^ F-FDG-PET/CT with dual time point imaging. The sternal FDG uptake was assessed visually (as positive or negative) and metrically using the maximum standardized uptake values (SUV _max_ ) calculated automatically. The SUV _max_ was compared between the patients with and without the use of bone wax and among patients with and without positive sternal findings in the visual analysis. A correlation analysis was performed between the time since surgery and the sternal SUV _max_ .

**Results:**

In all eight patients (32%) had received bone wax. In those patients, the mean sternal SUV _max_ was higher compared to the group without bone wax, both in the early (4.74 ± 1.28 vs. 3.70 ± 1.44; *p* = 0.0969) and in the late images (6.62 ± 2.67 vs. 4.36 ± 1.44; *p* = 0.0289). Moreover, the use of bone wax was strongly associated with positive sternal findings in the visual analysis (OR = 10; 95%CI = 0.995–100.462; *p* = 0.0421). The correlation analysis revealed a slightly decreasing trend without significance (Spearman's ρ = −0.139; *p* = 0.505).

**Conclusion:**

The use of bone wax during sternotomy could be associated with increased sternal uptake of FDG on ^18^ F-FDG-PET/CT, even several years after surgery. This finding should be considered in the evaluation of potential deep sternal wound infections.

## Introduction

Deep sternal wound infection (DSWI) as well as infective sternal osteomyelitis are rare complications after median sternotomy during open heart surgery. These serious medical conditions are potentially life-threatening and usually require surgical debridement. Therefore, the early and precise diagnosis of DSWI is crucial for the prognostic outcome of the patients. In general, the diagnosis of DSWI is based on clinical features such as purulent or bloody discharge from the wound area, chest pain, sternal instability, fever, other local signs of infection, or increased infection parameters in the patients' blood like leukocytosis, elevated C-reactive protein (CRP), or procalcitonin as well as positive microbiological results from swabs taken from the sternal wound. 1 In some cases, further imaging is needed to confirm suspected infections. Apart from the conventional imaging techniques, ^18^ F-FDG-PET/CT has been demonstrated as a highly sensitive imaging modality in the detection of infectious lesions. In detail, ^18^ F-FDG-PET/CT visualizes increased inflammation related glucose metabolisms and is associated with both high sensitivity and high specificity in the detection of DSWI. 2 3 However, physiologic healing processes following sternotomy can mimic infective FDG uptake enhancement of the bone tissue and therefore complicate the interpretation of the images. Indeed, increased FDG uptake of the sternum is common after recent sternotomy, even in non-infectious patients. 3 Although the intensity of sternal FDG uptake decreases over time, persistently elevated uptake findings have recently been described in up to 45% of noninfectious patients even 1 year after surgery. 4

In addition, several surgical procedures have been associated with artificially increased uptake findings on ^18^ F-FDG-PET/CT imaging, such as foreign body reactions to hemostatic materials or surgical adhesives. 5 6 In this context, the metabolic presentation of bone wax applied during skeletal surgery has not been investigated so far.

The use of bone wax during open heart surgery is a common intraoperative procedure for the control of osseous bleedings arising from thoracotomy. Besides periosteum, cancellous bone is the main source of bleeding. Since cauterization is not useful in the latter, bone wax can be applied to the sternal cut surfaces to prevent further bleeding. It consists of bee wax and isopropylpamitate with intention to reduce the risk of wound infections. However, nothing is known about the metabolic behavior or the surrounding tissue reaction to this material in the long term. Therefore, the aim of our study was to investigate the potential impact of sternal bone wax application during cardiac surgery on ^18^ F-FDG-PET/CT imaging to prevent false positive diagnosis of misinterpreted DSWI in the future.

## Methods

### Patients and Data Collection

All patients who had a No-React Aortic BioConduit (BioIntegral Surgical Inc., Mississauga, Canada) implanted at our center between January 2014 and April 2022 were screened for the study. Of these 25 patients, operated on between February 2018 and April 2022, were still alive and were willing to take part. Only patients with no prior evidence of acute endocarditis were included. Minors and patients who had undergone complete aortic root replacement surgery less than 3 months previously were excluded. All patients had received a replacement of the aortic valve and the ascending aorta using a No-React Aortic BioConduit. The surgical procedures were carried out between February 2018 and April 2022. Detailed characteristics of the patient cohort are presented in Table 1 . In nine patients, this was the first cardiac surgery. Prior to this surgery, 15 patients had received a prosthetic aortic valve or a replacement of the ascending aorta and 1 patient underwent cardiac surgery two times before the last intervention.

**Table 1:** Detailed characteristics of the patient cohort ( *n* = 25)

	Mean	SD	Median	Range
Age	63.20 years	± 9.66 years	62 years	47–83 years
Time since surgery	3.05 years	± 1.30 years	2.60 years	1.32–5.48 years
Height	1.81 m	± 0.08 m	1.83 m	1.58–1.96 m
Weight	89.24 kg	± 17.32 kg	85 kg	63–120 kg
BMI	27.23 kg/m ^2^	± 4.40 kg/m ^2^	25.98 kg/m ^2^	19.73–36.30 kg/m ^2^
Blood glucose level	100.96 mg/dL	± 17.93 mg/dL	99 mg/dL	79–167 mg/dL
Activity applied	267.32 MBq	± 46.31 MBq	262 MBq	195–351 MBq
Imaging delay (early)	65.48 minutes	± 9.93 minutes	65 minutes	51–95 minutes
Imaging delay (late)	123.45 minutes	± 10.97 minutes	121.50 minutes	105–149 minutes
**Reason for surgery**		**Number of patients**		
Native aortic valve insufficiency		8/25 (32%)		
Native aortic valve stenosis		3/25 (12%)		
Aortic dissection		3/25 (12%)		
Aortic aneurysm		8/25 (32%)		
Prosthetic valve endocarditis		11/25 (44%)		

Abbreviations: SD, standard deviation.

Notes: Range = minimum to maximum. Height, weight, BMI, and blood glucose level measured on examination day. The proportion of patients (reason of surgery) exceeds 100% due to partially combined pathologies.

All patients underwent ^18^ F-FDG-PET/CT imaging between June 2023 and August 2023. On the examination day, transthoracic echocardiography was performed prior to the PET/CT examination by a specialist in cardiology. Moreover, standardized blood samples were taken both for microbiological culturing and for laboratory analyses. In detail, measured laboratory data included full blood count, renal function parameters (creatinine, glomerular filtration rate), and CRP values. The time elapsed between last cardiac surgery and the PET/CT examination day was documented and used for further analyses.

### PET/CT Imaging

All patients were asked to maintain a low-carb-high-fat diet for 2 days prior to the examination with the intention to suppress the physiologic myocardial glucose metabolism as described elsewhere. 7 In preparation for the PET/CT scan, fasting for at least 6 hours before the injection of the radionuclide was required.

After measuring capillary blood glucose levels, ^18^ F-FDG (Eckert & Ziegler, Berlin, Germany) was injected intravenously. After approximately 60 minutes, a standardized PET/CT scan of the trunk was performed using a Biograph mCT 40 PET/CT scanner (Siemens Healthineers, Erlangen, Germany), followed by a late imaging sequence of the same region after approximately 120 minutes. In three cases, no late sequence was acquired due to technical issues or refusal of the patient.

The acquisition protocol corresponded to the EANM and SNMMI recommendations for the use of 18F-FDG-PET in infections. 8

### Analysis of Findings

**Fig. 1:**
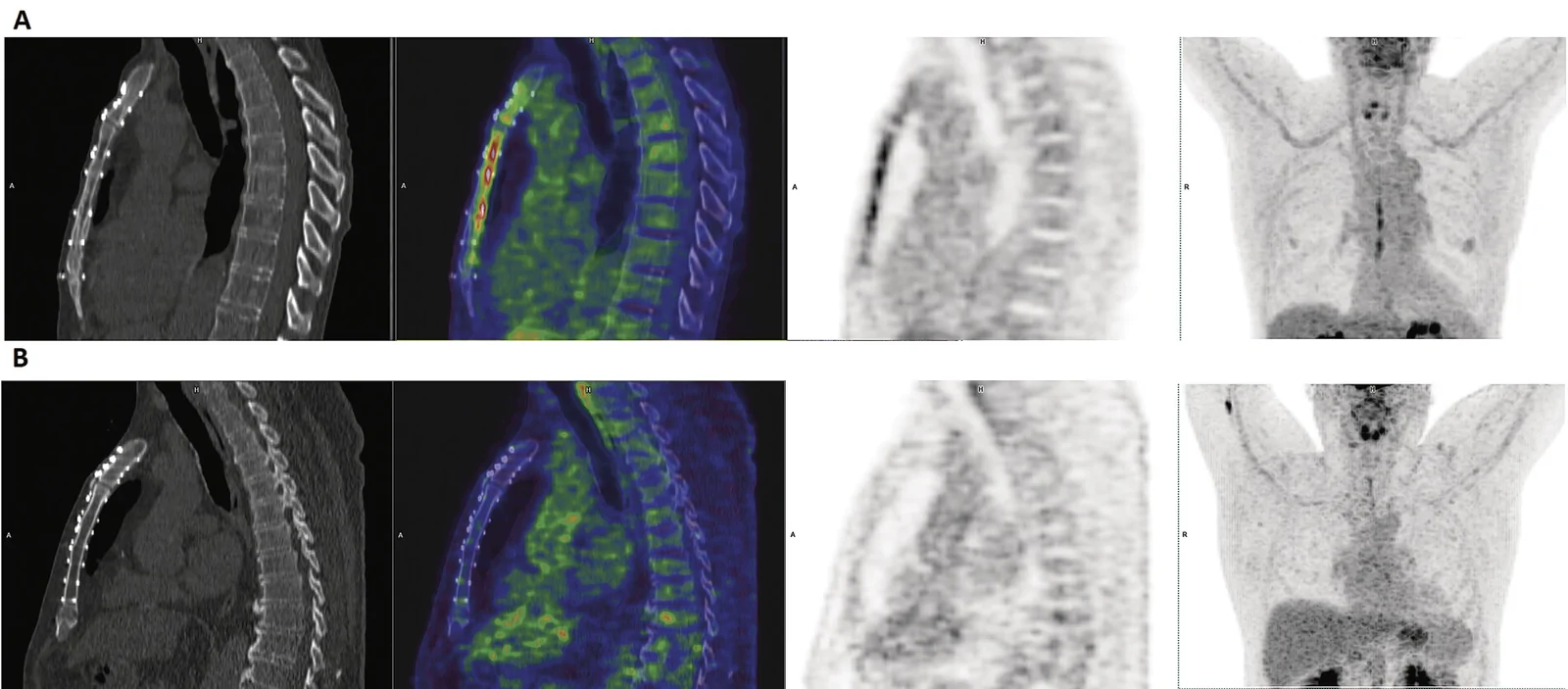
Examples of visually negative and visually positive sternal findings. Sagittal slices (from left to right: CT, PET/CT, PET) and maximum intense projection (MIP) in anterior view (right): Upper row (A): Positive finding in a 71 years old male, 2.1 years after sternotomy (bone wax positive). Lower row (B): Negative finding in a 55 years old male, 2.4 years after sternotomy (bone wax negative).

Post-processing and analyzing of the images were performed by two specialists in nuclear medicine and radiology using a Syngo.via workstation (Siemens Healthineers, Erlangen, Germany). Regions of interest (ROIs) were drawn around the sternum, around the prosthetic ascending aorta, and the aortic valve. The uptake of the sternum was assessed visually and by maximum standardized uptake values (SUV _max_ ) calculated automatically. The visual evaluation was performed in consensus of the two above-mentioned board-certified specialists. For visual assessment, the uptake was divided into three groups: Negative (sternal uptake < thoracic vertebral uptake), slightly positive (sternal uptake slightly above the thoracic vertebral uptake), and strongly positive (sternal uptake strongly above the thoracic vertebral uptake). For further analyses, the individuals with slightly positive and strongly positive findings were taken together to a combined group defined as positive. Examples of visual positive and visual negative findings are presented in Fig. 1 .

### Statistical Analysis

Nonparametric rank correlation analysis of the clinical parameters and the SUV _max_ values was performed using Spearman's method.

Cross table results were analyzed with calculation of the odds ratio and Fisher's exact test.

Differences of the mean were tested using nonparametric Mann-Whitney U test.

The significance level was set to 0.05. All statistical calculations and figures were performed/created using the Matlab R2023b software (MathWorks Inc., Natick, USA).

### Ethics

The study was performed in accordance with the ethical standards laid down in the 1964 Declaration of Helsinki and its subsequent revisions and has been approved by the institutional review board (AZ: D504/22). Written informed consent was obtained from all subjects.

## Results

**Fig. 2:**
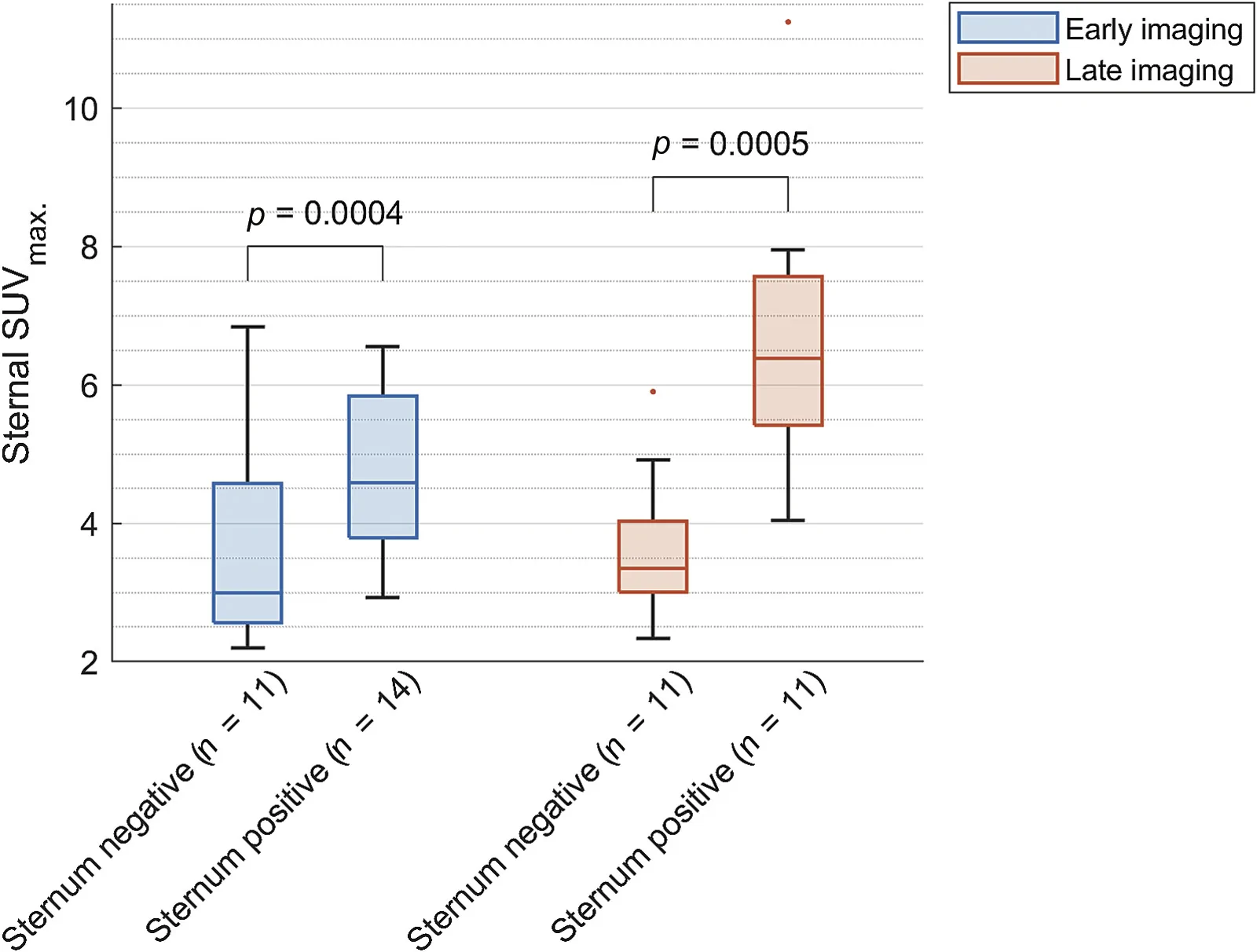
SUV _max_ of visually negative and visually positive individuals. In three cases, there was no late imaging available. All of these patients were found to be visually positive in the early scan. *p* -value calculated by Mann-Whitney U test. The whiskers indicate the 1.5-fold interquartile range.

In the visual analysis, 14 out of 25 patients (56%) were found to have positive sternal findings in the ^18^ F-FDG-PET/CT imaging (11 patients strongly positive, 3 patients slightly positive), both in the early and in the late scan. Detailed findings are presented in Table 2 . Comparing the SUV _max_ values between the visually positive and the visually negative patient groups, there was a statistically significant difference of the means in the early images (4.89 ± 1.08 vs. 2.95 ± 1.10; median 4.67 vs. 2.68; *p* = 0.000414, see Fig. 2 ). In the analysis of the late PET/CT images, the difference of the means was even more robust (6.52 ± 2.01 vs. 3.64 ± 1.02; median 6.38 vs. 3.35; *p* = 0.000501).

**Table 2:** PET/CT parameters and laboratory findings

	Overall	Bone wax positive	Bone wax negative	Visually positive	Visually negative
Number of patients	25	8	17	14	11
Years since surgery	3.05 ± 1.30	3.28 ± 1.50	2.93 ± 1.22	3.09 ± 1.31	2.99 ± 1.35
Age (years)	63.20 ± 9.66	63.88 ± 10.33	62.88 ± 9.64	65.57 ± 10.01	60.18 ± 8.70
Sternal SUV _max_ early	4.03 ± 1.45	4.74 ± 1.28	3.70 ± 1.44	4.89 ± 1.08	2.95 ± 1.10
Sternal SUV _max_ late a	5.08 ± 2.14	6.62 ± 2.67	4.36 ± 1.44	6.52 ± 2.01	3.64 ± 1.02
CRP (mg/L)	11.47 ± 19.41	15.69 ± 22.55	9.48 ± 18.15	16.10 ± 24.58	5.57 ± 7.06
Leukocyte count/nL	7.08 ± 2.44	7.90 ± 3.05	6.70 ± 2.09	6.86 ± 2.46	7.37 ± 2.51
GFR (mL/min)	72.96 ± 21.34	68.25 ± 17.34	75.18 ± 23.13	75.21 ± 18.17	70.09 ± 24.46

Abbreviations: CRP, C-reactive protein; GFR, glomerular filtration rate; SUV _max_ , maximum standardized uptake values.

Notes: Data given as mean ± standard deviation.

a Since there were no late series in three patients, the given data are calculated for *n* = 22 (overall), *n* = 6 (bone wax positive), *n* = 16 (bone wax negative), n = 11 (visually positive), *n* = 11 (visually negative) patients.

Overall, bone wax was applied in 8 out of 25 patients (32%) during surgery. Considering these patients, there was a strong disbalance of visually positive ( *n* = 7) and visually negative ( *n* = 1) patients. In contrast, the patient group without applied bone wax showed an even more balanced distribution with 7 visually positive and 10 visually negative individuals. Indeed, the odds ratio proved a strong association of positive sternal findings with the use of bone wax (OR = 10; 95% CI = 0.995–100.462; *p* = 0.0421; see crosstable in Table 3 ).

**Table 3:** Crosstable

Number of patients (percentage)	Bone wax positive	Bone wax negative	Total	OR	95% CI	*p*
Positive sternal findings	7 (50%)	7 (50%)	14 (100%)	10	0.995–100.462	0.0421
Negative sternal findings	1 (9 %)	10 (91%)	11 (100%)			

Abbreviations: 95% CI, 95% confidence interval; OR, odds ratio.

Note: *p* calculated with Fisher's exact test.

**Fig. 3:**
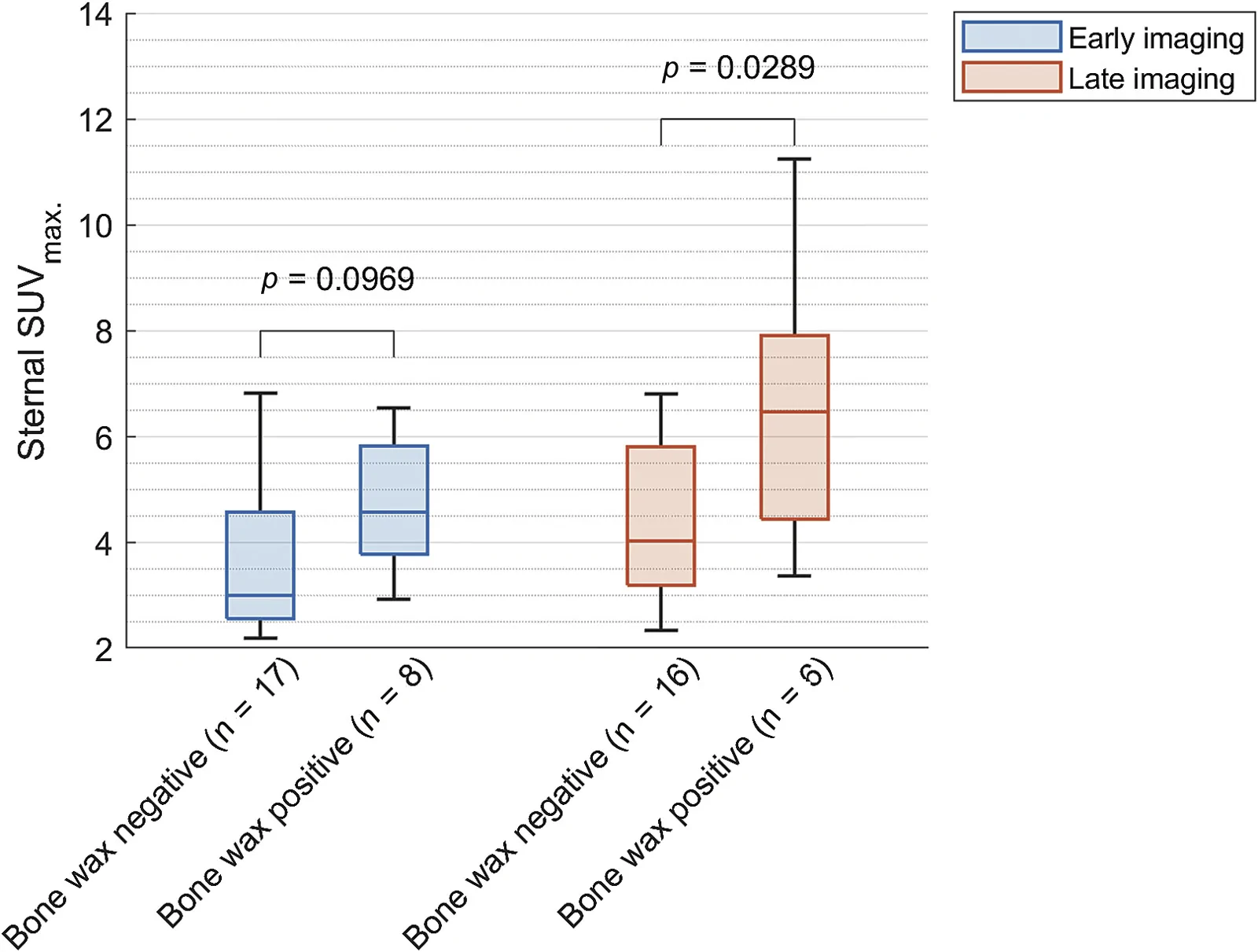
SUV _max_ of bone wax negative and bone wax positive individuals. In three cases, there was no late imaging available. Out of these patients, two patients were bone wax positive and one patient was bone wax negative. *p* -value calculated by Mann-Whitney U test. The whiskers indicate the 1.5-fold interquartile range.

The mean of the sternal SUV _max_ differed between the patient group with applied bone wax (4.74 ± 1.28; median 4.58) and the patient group without the use of bone wax (3.70 ± 1.44; median 3.00) in the early PET/CT images, but this difference did not remain significant in the statistical analysis ( *p* = 0.0969). However, the discrepancy of the means was even more pronounced in the late PET/CT images (6.62 ± 2.67 vs. 4.36 ± 1.44; median 6.47 vs. 4.04; *p* = 0.0289). The distribution of the SUV _max_ between the two groups is shown in Fig. 3 .

**Fig. 4:**
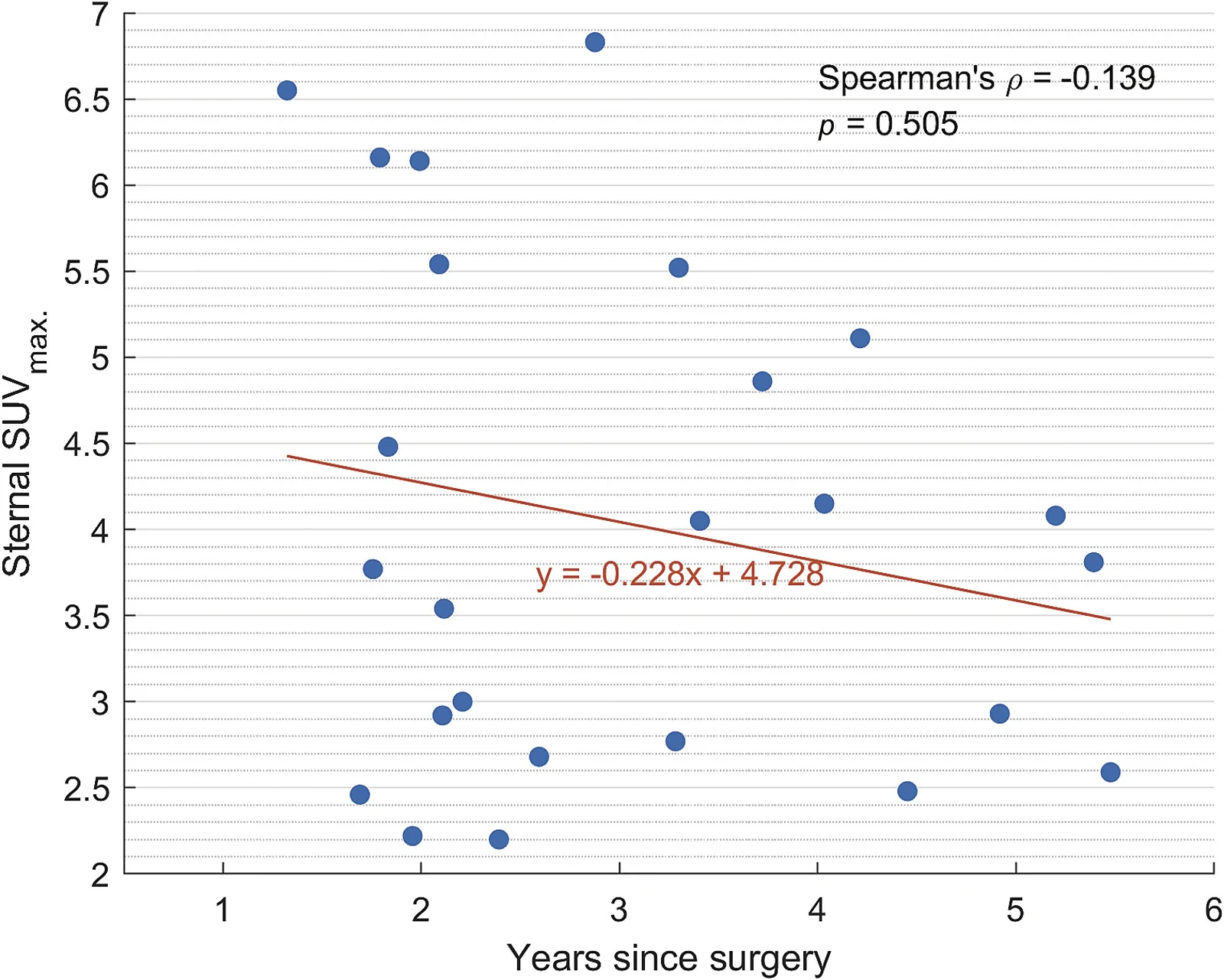
Sternal SUV _max_ in the early PET/CT images in dependence of SUV _max_ on time since surgery.

In the correlation analysis of the whole patient group, there was a slightly decreasing trend of the sternal SUV _max_ in association with time elapsed since surgery without statistical significance in the early images (Spearman's ρ = −0.139; *p* = 0.505) but not in the delayed images (Spearman's ρ = 0.067; *p* = 0.766). A more detailed distribution of the early SUV _max_ values in dependence of SUV _max_ on time since surgery is shown in Fig. 4 .

There were no associations between the laboratory findings (i.e. CRP, leukocytes, renal function parameters, blood cultures) and the PET/CT findings, neither in the visual assessment nor in the SUV _max_ measurement (data not shown). Only one patient had leukocytosis and one patient was found to have *E. coli* (in 5/6 samples) and *S. hominis* (in 1/2 samples) in blood cultures, which was related to an acute spondylodiscitis (confirmed by intraoperative swab specimen). All other laboratory findings were unremarkable. Similarly, the transthoracic echocardiography was unremarkable in all patients.

## Discussion

In this study, we aimed to assess the sternal FDG uptake on ^18^ F-FDG-PET/CT after the use of bone wax to achieve hemostasis during sternotomy. First of all, we must emphasize that the study's conclusions should be interpreted cautiously due to the relatively small sample size of patients involved. Nevertheless, due to the significant differences between the two groups, it can be concluded that there is an association of an increased sternal FDG uptake and the use of bone wax, both in the absolute SUV _max_ values and in the 10-fold increased odds ratio. This finding is even more emphasized by the relatively small patient cohort. However, this substantial result might be diminished by the statistical result of the 95% CI which overlaps from 100.462 to 0.995. Formally, the lower limit of the CI should be >1 to reach full statistical validity. However, since the lower limit nearly equals 1, we think that the calculated odds ratio can still be assumed as a reliable association.

The increased metabolic activity of sternal bone wax in the 18F-FDG-PET/CT imaging more than 1 year after the surgical procedure is presumably caused by sterile inflammatory processes. Since bone healing is generally completed after 3 to 6 months, the 18F-FDG-PET/CT examination should show normal findings. Indeed, bone wax is known to induce chronic inflammatory reactions. 9 For instance, the use of bone wax has been associated with reduced bone healing in sternotomized goats, 10 with foreign-body reactions and lack of bone reformation of surgical defects in the iliac crest of dogs 11 and with tissue reactions to cranial bone defects in rabbits. 12 In very rare cases, even the formation of bone wax granulomas and tumors has been reported in the long-term observation. 13 14

Given these reports, chronic inflammatory reactions to bone wax remnants left in the sternum after thoracotomy appear to be a plausible reason for increased sternal FDG uptake on PET/CT imaging.

Interestingly, all demonstrated effects were more pronounced in the late images than in the early images, although the mean metabolic activity of the sternum raised even in the visually negative patients. This finding is well in line with another description of increasing metabolic activity in the normal bone marrow over 3 hours after injection of the radionuclide on ^18^ F-FDG-PET/CT. 15 However, dual time point ^18^ F-FDG-PET/CT imaging is commonly used to differentiate between malignant and benign or inflammatory findings, whereby the FDG uptake in inflammatory processes usually decreases or remains stable in the late time points. 16 Especially in chronic bacterial osteomyelitis, one study demonstrated a consistent trend in 16 patients with a median reduction of the SUV _max_ in the infected tissue by 6% in the late images. 17 Remarkably, one patient in this cited study from Sahlmann et al showed a contrasting increase of the SUVmax over time. 17 In that case, multiple foreign body granulomas in addition to a mononuclear infiltrate were proven in the histological examination. Despite the fact that this finding is only limited transferable to our setting, this could be a hint that foreign body reactions might induce an elevated glucose metabolism with increasing activity in the late ^18^ F-FDG-PET/CT images.

Overall, one patient was found to have bacteremia with *E. coli* and *S. hominis* , which was related to an acute spondylodiscitis due to *E. coli* , whereas the detection of *S. hominis* was interpreted as contamination. Blood cultures of all other patients remained negative after 7 days of bacteriological incubation. Additionally, the leukocyte count was found to be normal in 24/25 patients. Therefore, we deduce that the remarkable FDG enhancement in the sternum of the visually positive patients is least likely related to an infective reason.

Our result of 56% positive sternal findings is consistent with other reports in the literature. A recently performed study with 44 individuals demonstrated a continuous reduction of the sternal SUV _max_ from 7.34 to 3.20 during the first year after sternotomy with persistently elevated uptake patterns in up to 45% of the patients at 55 weeks after surgery. Following these findings, our data did not show a further decrease of the sternal activity over the next 6 years. This might indicate that the healing process is mostly completed after 1 year. However, patients with a history of sternotomy within the first year prior to the PET/CT imaging were not included in our study. In another study for the evaluation of the diagnostic performance of ^18^ F-FDG-PET/CT in the detection of DSWI, increased focal uptake was observed in 72% of the non-infectious control group of 29 individuals who were examined at a mean of 42.4 months after sternotomy. 3 Complementary to those findings, there was no relevant association of the time since surgery with the metabolic activity of the sternum in our study too. However, the use of bone wax was not considered in the studies mentioned above.

Nevertheless, there are some limitations of our study. First, we assume a noninfected state of the sternum in our patients based on clinical and laboratory findings, but a chronic osteomyelitis cannot be definitively ruled out. We didn't have a control group with histopathological or microbiologically proven sternal osteomyelitis for comparison. The patient number who received bone wax is small. Therefore, further studies with larger patient cohorts are necessary to confirm our hypothesis. However, the strong effect size of our findings is even more emphasized by the small patient group.

## Conclusion

The use of bone wax during sternotomy could be associated with increased sternal uptake of FDG on ^18^ F-FDG-PET/CT, even several years after surgery. Thus, ^18^ F-FDG-PET/CT images for the evaluation of potential sternal osteomyelitis should be interpreted carefully to rule out false positive results, particularly when bone wax hemostasis was used during surgery.
